# Mechanics Model for Simulating RC Hinges under Reversed Cyclic Loading

**DOI:** 10.3390/ma9040305

**Published:** 2016-04-22

**Authors:** Ahmad Azim Shukri, Phillip Visintin, Deric J. Oehlers, Mohd Zamin Jumaat

**Affiliations:** 1 Department of Civil Engineering, University of Malaya, Kuala Lumpur 50603, Malaysia; ahmadazimshukri@gmail.com (A.A.S.); zamin@um.edu.my (M.Z.J.); 2 School of Civil, Environmental and Mining Engineering, The University of Adelaide, Adelaide, South Australia 5005, Australia; doehlers@civeng.adelaide.edu.au

**Keywords:** cyclic loading, RC beams, tension stiffening, concrete softening, hinge length

## Abstract

Describing the moment rotation (M/θ) behavior of reinforced concrete (RC) hinges is essential in predicting the behavior of RC structures under severe loadings, such as under cyclic earthquake motions and blast loading. The behavior of RC hinges is defined by localized slip or partial interaction (PI) behaviors in both the tension and compression region. In the tension region, slip between the reinforcement and the concrete defines crack spacing, crack opening and closing, and tension stiffening. While in the compression region, slip along concrete to concrete interfaces defines the formation and failure of concrete softening wedges. Being strain-based, commonly-applied analysis techniques, such as the moment curvature approach, cannot directly simulate these PI behaviors because they are localized and displacement based. Therefore, strain-based approaches must resort to empirical factors to define behaviors, such as tension stiffening and concrete softening hinge lengths. In this paper, a displacement-based segmental moment rotation approach, which directly simulates the partial interaction behaviors in both compression and tension, is developed for predicting the M/θ response of an RC beam hinge under cyclic loading. Significantly, in order to develop the segmental approach, a partial interaction model to predict the tension stiffening load slip relationship between the reinforcement and the concrete is developed.

## 1. Introduction

The ability of a reinforced concrete (RC) member to maintain load and rotate under increasing deflections—that is, member ductility—is vital to its ability to absorb energy inputs, such as those from seismic and blast loads. The importance of defining member ductility has made it a significant area of research since the 1960s and a substantial amount of experimental research to describe the hysteretic moment rotation (M/θ) behavior of RC members at all load levels has been performed. From this experimental research, it has been shown that the loss of stiffness associated with the cyclic loading of RC members arises due to the Baushinger effect, which describes the softening behavior of steel following reversal of load; concrete cracking and splitting along the reinforcement; cyclic deterioration of bond between the reinforcement and the concrete; and shear crushing and sliding of the concrete [1]. This implies that the behavior of reinforced concrete under cyclic loading is dominated by localized partial interaction (PI) behaviors.

In the tension region, this PI behavior of slip along the concrete to reinforcement interface is defined by the bond properties between the reinforcement and the concrete [2,3,4] and is responsible for crack development and crack widening and closing [5,6,7,8,9,10,11,12,13,14,15], as well as sliding of the concrete [16,17].

In order to simulate the behavior seen in practice it is, therefore, necessary to simulate these localized partial interaction behaviors. Typical strain-based moment curvature approaches cannot directly simulate localized PI displacements and, hence, must rely heavily on empiricisms, such as the use of effective flexural rigidities to simulate tension stiffening [18,19,20,21,22] and hinge lengths [23,24,25,26,27,28] to quantify hinge rotations. The challenges of modelling localized deformations also exist in finite element analyses where localizations are generally either modelled through the smearing of cracks and, hence, do not capture the behavior seen in practice, or though discrete crack modelling using automatic remeshing, for which analysis with complex crack patterns remains a challenge [15,29,30].

The use of standard strain-based moment curvature analysis procedures is, therefore, generally limited, in that the behavior seen in practice—that is, the formation, widening and closing of cracks in the tension region and the formation and failure of concrete softening wedges in the compression region—is not directly simulated. Moreover, the empirical factors applied to simulate the localized partial interaction behaviors are limited in use to within the bounds from which they were derived and correlation outside these bounds is often poor [28]. In response to the limitations imposed by these empiricisms, the authors developed a moment-rotation (M/θ) approach which directly simulates the slip between reinforcement and the concrete using partial interaction theory, as well as the formation and failure of concrete softening wedges using shear friction theory.

For members with “weak” bond—that is, for members where debonding of the reinforcement defines failure such as fiber reinforced polymer (FRP) plated members—the M/θ approach has been developed and applied to simulate both monotonic [9,31] and cyclic [32] loading. For members with a “strong” bond between the reinforcement and concrete, such as members reinforced with ribbed steel reinforcement where reinforcement does not debond, a segmental M/θ approach has been developed to simulate the behavior of a segment between two cracks under monotonic loading [12,33]. In this paper, the segmental M/θ approach is extended to allow for the simulation of beams under cyclic loading. Hence, in this paper, the generic approach for applying a segmental analysis is presented. This is followed by the development of a new cyclic tension stiffening model which can be applied at all load levels and under reversal of loads, provided debonding does not occur. The segmental approach is then extended specifically to cyclic loading and consideration is made to the incorporation of concrete softening through the use of a size dependent stress strain relationship.

This paper introduces a new tension stiffening model which allows for the formation and simulation of multiple cracks along the entire span of RC beams under cyclic loading. The segmental M/θ approach also includes the incorporation of a size-dependent stress-strain relationship for concrete under cyclic loading, which is used to model the concrete softening of RC hinges when under cyclic loading. The proposed segmental M/θ approach is then validated against experimental results.

## 2. Segmental Moment Rotation Approach

**Figure 1:**
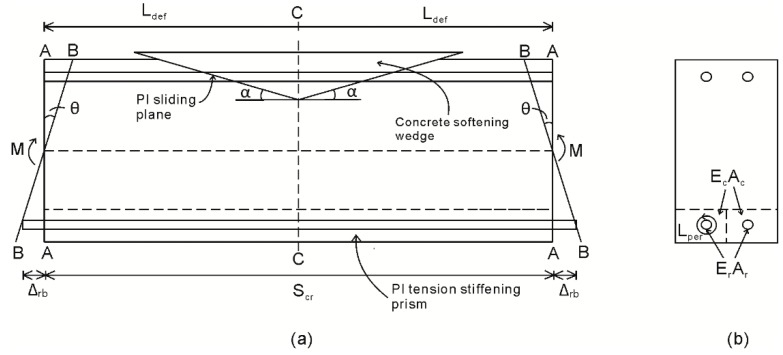
Segment of RC beam. (**a**) Beam segment; and (**b**) beam cross-section.

**Figure 2:**
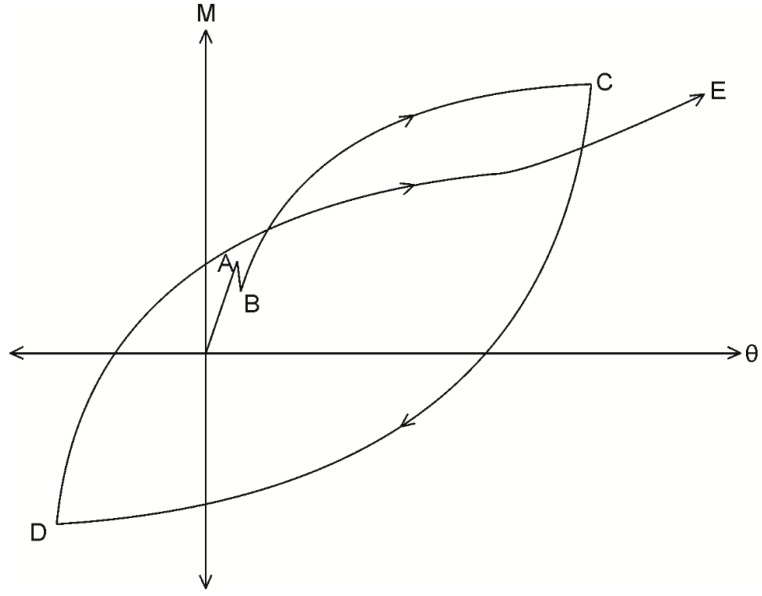
M/θ relationship.

Consider the segment shown in Figure 1a, which has a cross-section as shown in Figure 1b. The segment is extracted from an RC beam and is subjected a constant moment M which can be any moment in a cyclic load history. It is now required that the corresponding rotation at the segment ends θ be determined such that a single point within the moment rotation (M/θ) relationship, such as that in Figure 2, can be established.

The rotation θ at the segment ends in Figure 1a is accommodated by an Euler-Bernoulli deformation from A–A to B–B such that plane sections remain plane. The total deformation from A–A to B–B must be accommodated by a combination of material extension and contraction and non-material deformations due to sliding; that is, by a combination of material strains and localized partial interaction slip behaviors. In the tension region, a partial interaction slip between the reinforcement and the concrete, shown as Δ_rb_ in Figure 1a, takes place. This slip, and the associated loads in the reinforcement and concrete within the tension stiffening prism, defines the crack spacing, S_cr_ in Figure 1a, as well as the crack widths 2Δ_rb_ both during crack opening and closing, as well as tension stiffening. In the compression region, slip along a concrete to concrete PI sliding plane in Figure 1a results in the formation of concrete softening wedges seen in practice [12,14,15,33,34,35].

**Figure 3:**
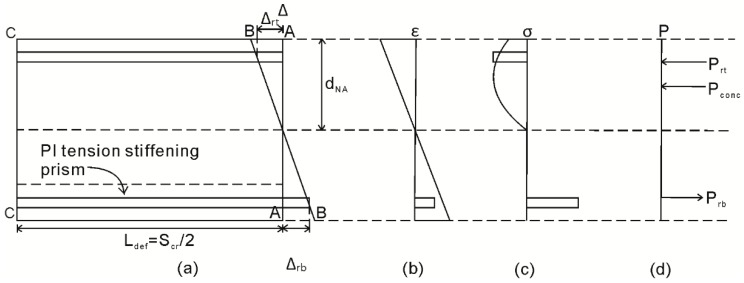
Half segment for analysis. (**a**) Half beam segment; (**b**) strain profile; (**c**) stress profile; and (**d**) load profile.

To describe how the segmental approach directly incorporates the simulation of those PI mechanisms described in the previous paragraph, let us consider one half of the segment now shown in Figure 3 [12,33]. It should be noted that, for analysis, only half the segment in Figure 1a which is of length L_def_ = S_cr_/2 is required due to symmetry around the center line C–C.

In Figure 3a, the deformation from A–A to B–B results in the strain profile in Figure 3b. This strain profile can be quantified by dividing the deformation from A–A to B–B by the length over which it must be accommodated; that is, L_def_. Prior to any strain localizations, and from the distribution of strain in Figure 3b, the distribution of stress in Figure 3c and the distribution of forces in Figure 3d can be determined from the material constitutive relationships. For the distribution of forces in Figure 3d, the neutral axis depth d_NA_ can be adjusted until, for a given rotation θ, longitudinal equilibrium is achieved. It can be noted that this analysis prior to strain localization provides identical results to a strain-based moment curvature analysis, as it is identical apart from the starting point of a displacement profile.

Let us now consider incorporation of the PI mechanism associated with concrete cracking [12,14,15,32,33] and the allowance of cyclic loading into this mechanism [32]. In Figure 3, following cracking and when the crack tip extends above the level of the tensile reinforcement, the load developed in the reinforcing bar P_rb_ is no longer a function of the linear strain profile in Figure 3b. Rather, the force P_rb_ in Figure 3d is a function of the slip of the reinforcement from the crack face Δ_rb;_ this can be determined through the application of partial interaction theory, which will here be extended to the cyclic load case to allow for tension stiffening. Importantly, the relationship between Δ_rb_ and P_rb_, as well as the strains, stresses and forces developed in the uncracked region in Figure 3 are a function of the deformation length L_def_ = S_cr_/2 and, hence, it is necessary to first establish crack spacing S_cr_.

**Figure 4:**
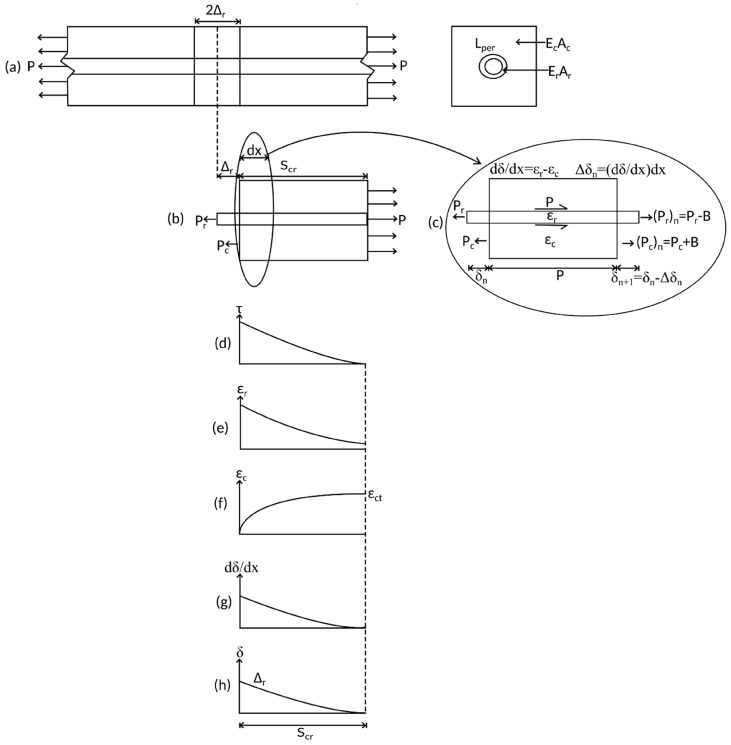
Partial-interaction tension stiffening analyses. (**a**) RC beam; (**b**) beam segment; (**c**) beam element; (**d**) bond stress distribution; (**e**) steel strain distribution; (**f**) concrete strain distribution; (**g**) slip-strain distribution; and (**h**) slip distribution.

Consider the PI tension stiffening region in Figure 1a now shown in Figure 4a. The prism has a cross-section where a single reinforcing bar of area A_r_ and moduli E_r_ is centrally located in a concrete prism of area A_c_ and moduli E_c_ such that when a load P_r_ is applied no moment is induced.

On initial axial loading P of the prism in Figure 4a that is prior to cracking, full interaction exists between the reinforcement and the concrete such that both the reinforcement and the concrete are uniformly extended. Following the formation of an initial crack, which occurs when the moment to cause cracking in the segment in Figure 1 is reached, a condition of partial interaction between the reinforcement and the concrete exists; that is, the reinforcement slips relative to the concrete resulting in a half crack opening Δ_r_ in Figure 4a,b.

**Figure 5:**
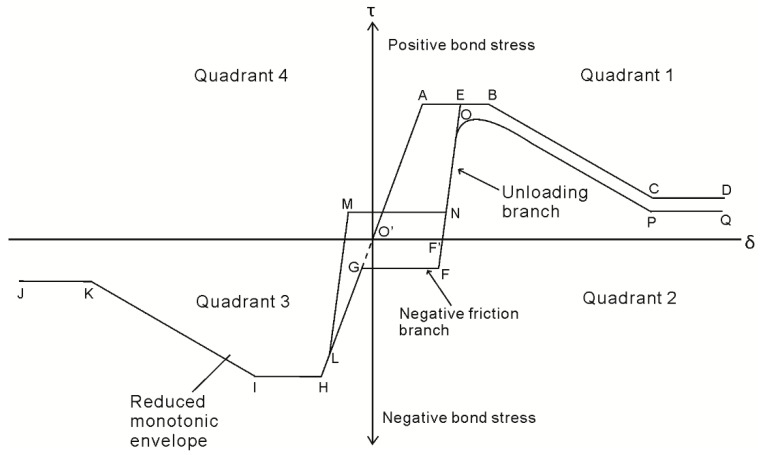
Cyclic bond stress-slip material properties.

For analysis [9,12,13,14,32,33] the prism in Figure 4b is discretized into elements of length dx where the first element along the bar length is shown in Figure 4c. The length dx should be small, say 0.1 mm such that it can be assumed that the stress and strain acting within the element can be considered uniform along length dx. Imposing a local slip of (δ_r_)_n_ on the element, which at the first element is equal to Δ_r_, the bond stress τ in Figure 4d is a function of the slip δ_r_ and is a material property which can be defined by testing. For example a schematic of the well-known model of Eligehausen *et al.* [4] is shown in Figure 5, where path OABCD defines the monotonic loading path which is required here to determine the crack spacing. Integrating the bond stress τ over the bonded area in Figure 4c that is L_per_dx, where L_per_ is the perimeter of the reinforcing bar, the bond force B for the element in Figure 4c is known.

An initial guess P_r_ for the load to cause slip Δ_r_ in Figure 4b is made and the load P_r_ is applied to the lefthand side of the first element in Figure 4c. From equilibrium, the force in the reinforcement at the righthand side of the element is P_r_-B. Similarly, taking the force in the concrete to be zero at a crack face, the force in the concrete at the righthand side is B. From material constitutive relationships and now knowing the average force in the reinforcement and the concrete in the element, the average strain in the reinforcement ε_r_ in Figure 4e and the concrete ε_c_ in Figure 4f is known. The slip strain dδ/dx in Figure 4g, which causes a change in slip over the element of length dx can then be defined as ε_r_ − ε_c_, where the change in slip over the element is Δδ_n_ = (dδ/dx)dx and, hence, the slip in subsequent elements can be determined. A shooting technique is then applied to determine the load P_r_ at which full interaction is achieved for a given slip Δ_r_ and this is obtained when the boundary condition δ = dδ/dx = 0 at the same location along the prism is met, as shown in Figure 4g,h. Applying the further condition that a subsequent primary crack will occur at the point of full interaction when the strain in the concrete reaches the tensile cracking strain in Figure 4f, the primary crack spacing can be determined [12,14,15].

**Figure 6:**
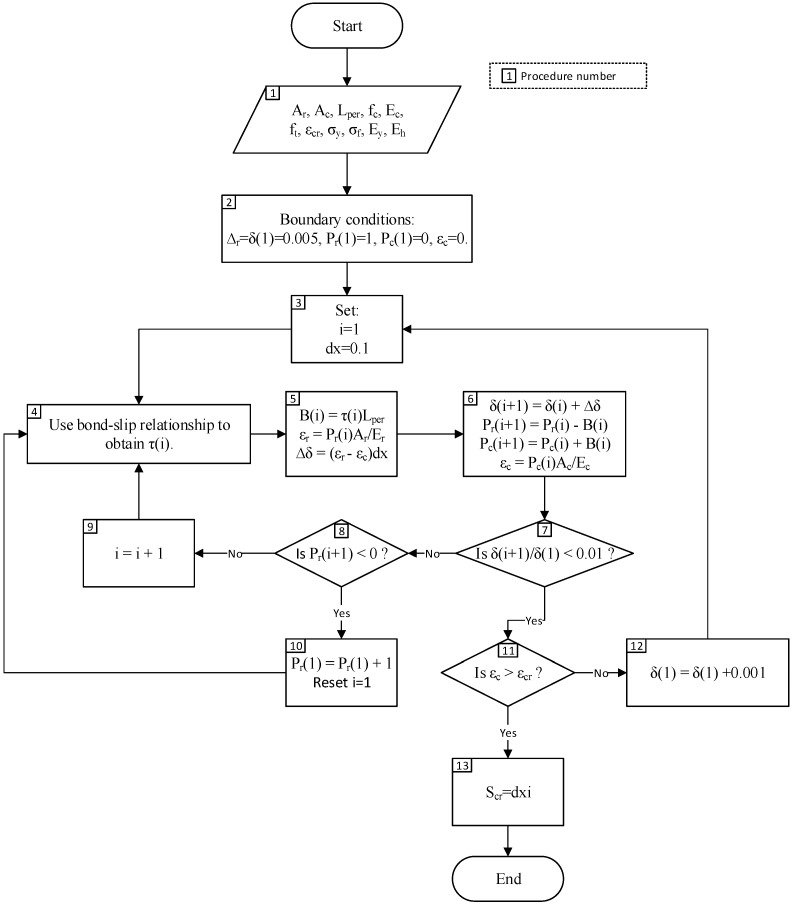
Procedure to determine primary crack spacing (S_cr_).

The numerical process to determine the primary crack spacing, S_cr_ is as shown below. The tension stiffening prism is first divided into small segments of length dx. A flowchart on the steps required is given in Figure 6. The analysis steps are as follows:

1. The required input data are inserted:
  - Area of steel reinforcement, A_r_.
  - Area of adjacent concrete, A_c_.
  - Perimeter of steel reinforcement, L_per_.
  - Concrete compressive strength, f_c_.
  - Concrete elastic modulus, E_c_.
  - Concrete tensile strength, f_t_.
  - Concrete cracking strain, ε_cr_ = f_t_/E_c_.
  - Yield strength of steel reinforcement, σ_y_.
  - Ultimate strength of steel reinforcement, σ_f_.
  - Elastic modulus of steel reinforcement, E_y_.
  - Strain hardening modulus of steel reinforcement, E_h_.
2. The first element is located at the crack face. Several boundary conditions are used:
  - The slip at the crack face, Δ_r_ = δ(1) = 0.005 mm.
  - Load applied on the adjacent concrete, P_c_(1) = 0 as the concrete-concrete interfaces are not touching at the crack face.
  - Load applied on steel reinforcement, P_r_(1) is assumed to be 1 N.
3. The dummy variable “i” is used to determine the number of the segment being solved. The initial value where i = 1 is used to determine the location of crack face, and larger values of i is the distance from the crack face. The length of one segment, dx = 0.1 mm.
4. Bond stress, τ(i) is determined using the bond-slip relationship from Comité euro-international du béton-Fédération Internationale de la Précontrainte (CEB-FIP) model code.
5. The bond force, which must be known to know how much load is transferred from the steel reinforcement to the adjacent concrete, is determined as B(i) = τ(i)L_per_. Strain of steel reinforcement is determined as ε_r_ = P_r_(i)A_r_/E_r_. The change in slip is then determined as ∆δ = (ε_r_ − ε_c_)dx.
6. With the value of B(i) and ∆δ determined, the values of boundary conditions for the next beam segment can be calculated. Note that the value of B(i) and ∆δ must be known for these to be calculated.
  - δ(i + 1) = δ(i) + ∆δ
  - P_r_(i + 1) = P_r_(i) − B(i)
  - P_c_(i + 1) = P_c_(i) + B(i)
  - ε_c_ = P_c_(i + 1)A_c_/E_c_
7. In practice it is not possible to reduce the slip completely to zero. As such the condition for full-interaction used is the reduction of slip such that δ(i + 1)/δ(1) < 0.01, which represents a 99% reduction from the original slip value at the crack face.
8. If the previous condition is not met, the location of full-interaction is still not met and another condition is checked, which is P_r_(i + 1) < 0.
9. If the previous condition is also not met, the analysis will be repeated for the next beam segment; the dummy variable i increased by 1 and Procedures 2–7 are repeated.
10. If the condition in Procedure 8 is met, the assumed value of applied load P_r_(1) is too low as the P_r_(i + 1) < 0 occurs prior to the full-interaction condition of δ(i + 1)/δ(1) < 0.01 is met. Procedures 2–7 are, thus, repeated with a higher value of assumed P_r_(1).
11. If Condition 7 is met, another condition is checked: ε_c_ > ε_cr_.
12. If Condition 11 is not met, the initial slip is too small to cause a primary crack and a larger value for the initial slip at the crack face, Δ_r_, will be set. The procedure 2–10 will then be repeated.
13. If Condition 11 is met, the primary crack has occurred. The required output from the analysis is the primary crack spacing which is determined as S_cr_ = dxi.

## 3. Cyclic Tension Stiffening between Cracks

**Figure 7:**
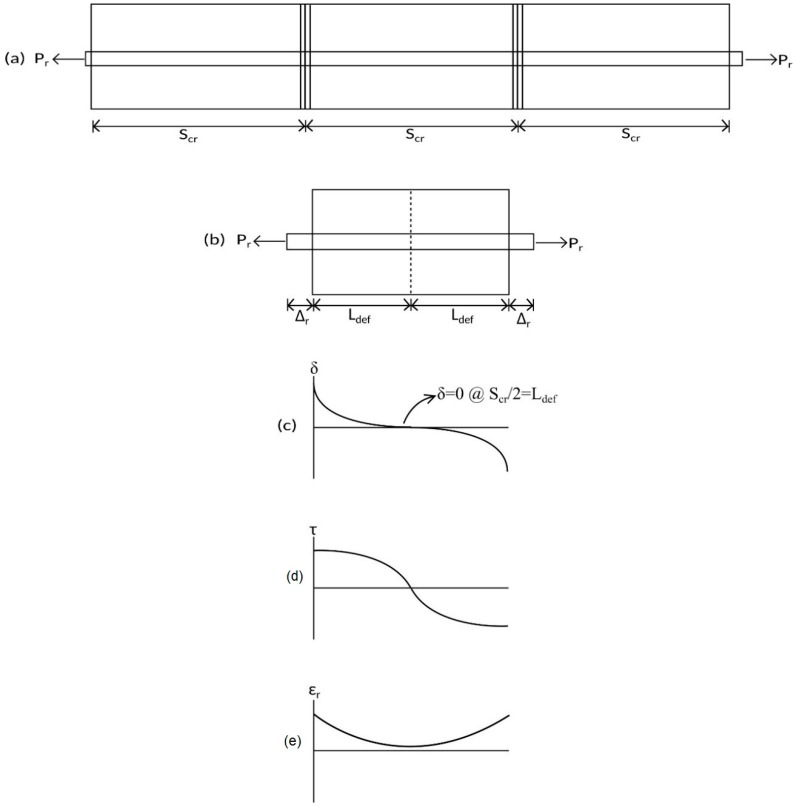
Tension stiffening prism during initial loading. (**a**) RC beam; (**b**) beam segment; and (**c**) slip; (**d**) bond stress; and (**e**) steel strain distribution.

Once primary cracks have formed, the PI mechanism changes from that in Figure 4a where a single crack is considered to that in Figure 7a where the prism has multiple cracks. Having been extracted from the constant moment region in Figure 1a, the prism is symmetrically loaded. Hence, as in Figure 7b, by symmetry the slip at the midpoint of each prism must be zero as in Figure 7c; that is, δ = 0 at S_cr_/2 from the crack face. This is the boundary condition for the symmetrically-loaded prism in Figure 7b which is in contrast to that with an initial crack in Figure 4b where the boundary condition is that of full-interaction.

**Figure 8:**
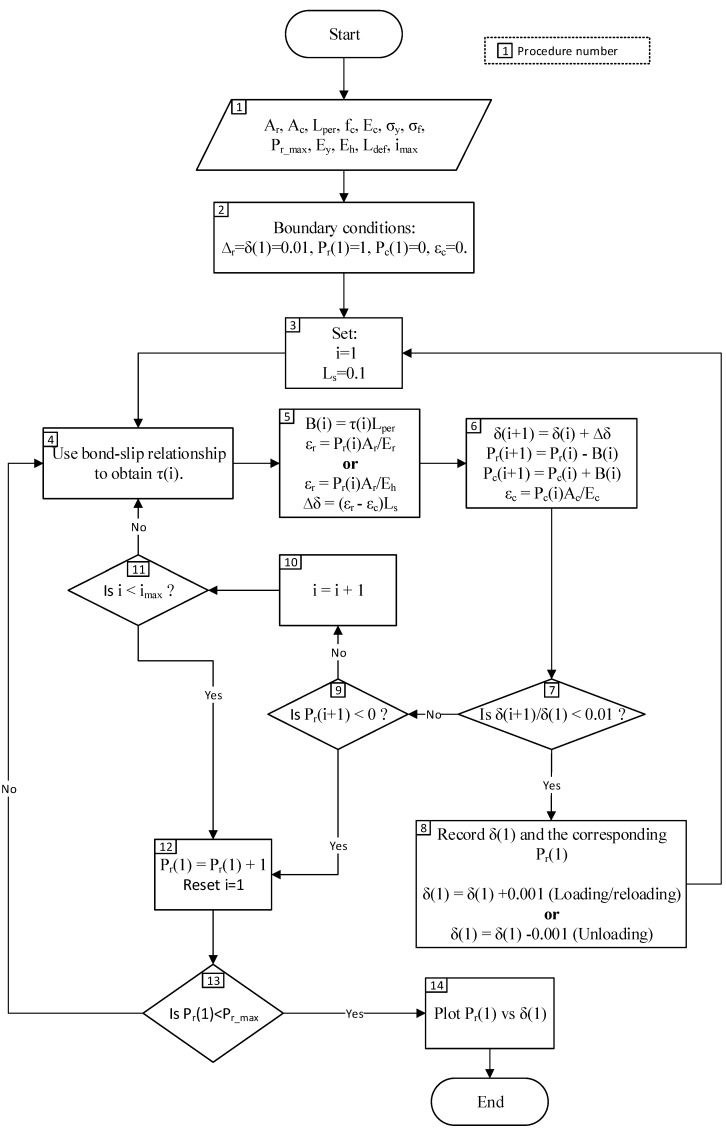
Procedure to determine the P_r_/Δ_r_ relationship.

It is now a matter of determining the relationship between the load P_r_ and the slip Δ_r_ shown in Figure 7 which is required for the segmental analysis in Figure 3. A flowchart on the steps required for the tension stiffening analysis is given in Figure 8. The analysis steps are as follows:

1. The required input data are inserted:
  - Area of steel reinforcement, A_r_.
  - Area of adjacent concrete, A_c_.
  - Perimeter of steel reinforcement, L_per_.
  - Concrete compressive strength, f_c_.
  - Concrete elastic modulus, E_c_.
  - Yield strength of steel reinforcement (if applicable), σ_y_.
  - Ultimate strength of steel reinforcement, σ_f_.
  - Ultimate load of steel reinforcement, P_r_max_ = A_r_σ_f_
  - Elastic modulus of steel reinforcement, E_y_.
  - Strain hardening modulus of steel, E_h_.
  - Length of deformation, L_def_ = S_cr_/2.
  - Number of elements, i_max_ = L_def_/dx.
2. The boundary conditions are used are:
  - The slip at the crack face, Δ_r_ = δ(1) = 0.01 mm.
  - Load applied on the adjacent concrete, P_c_(1) = 0 as the concrete-concrete interfaces are not touching at the crack face.
  - Load applied on steel reinforcement, P_r_(1) is assumed to be 1 N.
3. The variable i = 1 is used to determine the location of crack face, and larger values of i is the distance from the crack face. The length of one segment, dx = 0.1 mm.
4. Bond stress, τ(i) is determined using the cyclic bond-slip relationship from Eligehausen *et al.* [4].
5. The bond force is determined as B(i) = τ(i)L_per_. Strain of the reinforcement bar is determined as ε_r_ = P_r_(i)A_r_/E_r_. The change in slip is then determined as ∆δ = (ε_r_ − ε_c_)dx.
6. With the value of B(i) and ∆δ determined, the values of boundary conditions for the next beam segment can be calculated:
  - δ(i + 1) = δ(i) + ∆δ
  - P_r_(i + 1) = P_r_(i) − B(i)
  - P_c_(i + 1) = P_c_(i) + B(i)
  - ε_c_ = P_c_(i + 1)A_c_/E_c_
7. The condition for full-interaction used is the reduction of slip such that δ(i + 1)/δ(1) < 0.01, which represents a 99% reduction from the original slip value at the crack face.
8. If condition in Procedure 7 is met, the assumed value of applied load P_r_(1) is correct. The slip δ(1) and the corresponding P_r_(1) is then recorded and a larger/smaller value of δ(1) is set, depending on whether the beam is being loaded or unloaded.
9. If the condition in Procedure 7 is not met, the location of full-interaction is still not met and another condition is checked, which is P_r_(i + 1) < 0.
10. If the condition in Procedure 9 is also not met, the analysis will be repeated for the next beam segment; the dummy variable i increased by 1.
11. Another condition is then checked, which is i < i_max_ since the formation of primary cracks have limited the beam sections that are under partial interaction to half the crack spacing, also known as the length of deformation L_def_.
12. If condition in procedure 9 is met, the assumed value of applied load P_r_(1) is too low as the P_r_(i + 1) < 0 occurs prior to the full-interaction condition of δ(i + 1)/δ(1) < 0.01 is met. A higher value of P_r_(1) is thus assumed.
13. The P_r_(1) is checked whether it reaches or exceeds the ultimate load P_r_max_. If the condition is not met, procedures 4–13 is repeated.
14. If the condition in Procedure 13 is met, the steel reinforcement has fractured. The recoded values of Δ_r_ and P_r_(1) are then plotted to obtain the P_r_/Δ_r_ relationship.

The tension stiffening analysis presented in this research is considered to be an improvement to the previous research by the authors [32] which focused on quantifying the slip from a single hinge with the assumption that the member was rigid and all the rotations are lumped in this single hinge. The new tension stiffening analysis used in this paper does not use this simplification as the formation of primary cracks are simulated so that the member rotates along multiple cracks, thus allowing the curvature along the length of the beam to be correctly simulated. Due to the different boundary conditions the cyclic behavior of the tension stiffening prism simulated using the new tension stiffening analysis is significantly different from the previous work [32]. The new behavior of the prism will, hence, be presented in several stages beginning with that during initial loading.

### 3.1. Initial Loading

**Figure 9:**
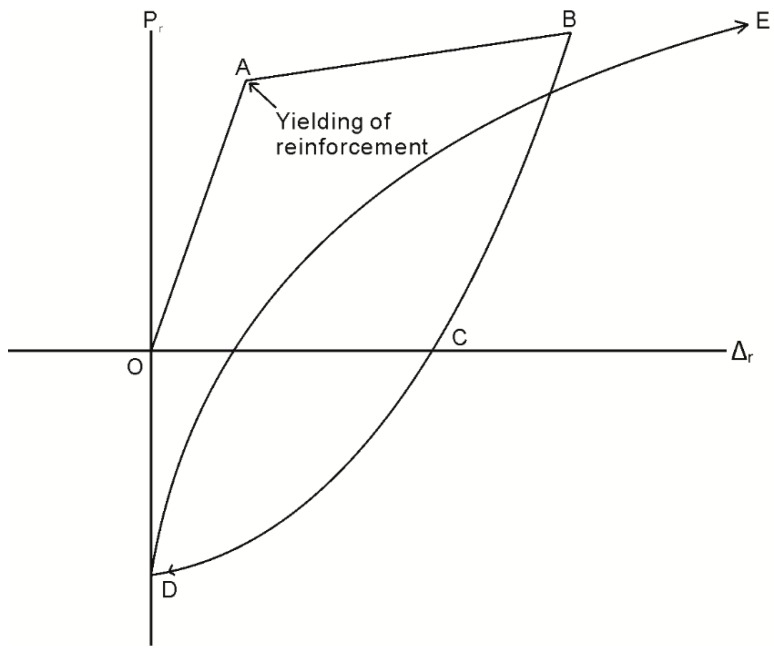
P_r_/Δ_r_ relationship.

During initial loading along path OAB in Figure 9, that is prior to any load reversals taking place, the same numerical analysis technique applied to determine the crack spacing can be applied; that is, a single element of length dx extracted from the prism in Figure 7b is identical to that in Figure 4c. The analysis, therefore, proceeds by imposing a slip Δ_r_ at the loaded end in Figure 7b and follows the same iterative procedure outlined in the previous section using Figure 4b,c except that the boundary condition is no longer that of full interaction but that the slip half way along the prism is zero and that δ = 0 at S_cr_/2, as shown in Figure 7c. From this analysis, for an imposed slip Δ_r_, the corresponding load P_r_, the distribution in slip in Figure 7c, the distribution of bond stress in Figure 7d, and the reinforcement strain in Figure 7e can be determined.

**Figure 10:**
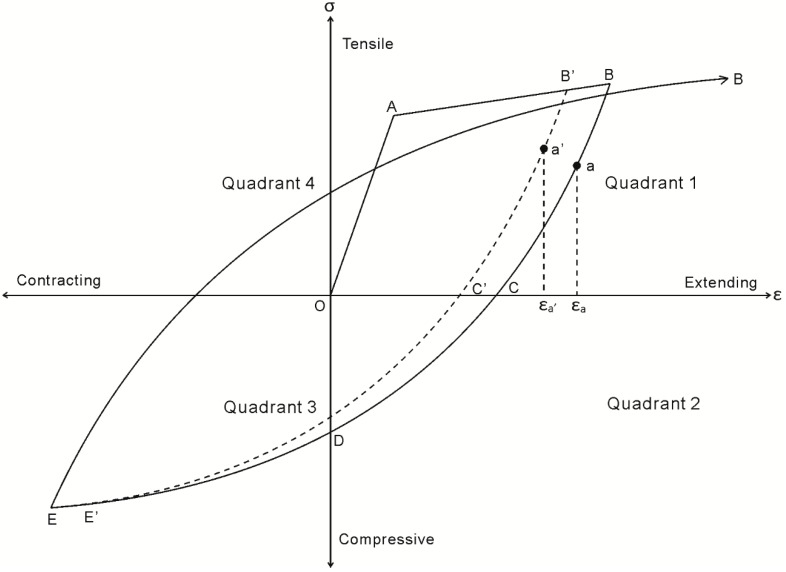
Cyclic stress-strain relationship of reinforcements.

Unique to the initial loading case, the material constitutive relationships are restricted to the monotonic behavior; that is, the bond τ/δ relationship as defined by path O’ABCD in Figure 5, and the reinforcing σ/ε relationship used to determine the reinforcement strain is restricted to path OAB in Figure 10. Upon unloading from point B in Figure 9, the P_r_/Δ_r_ relationship is strongly dependent on the load history of the local τ/δ bond properties, as well as the σ/ε relationship of the reinforcement. This dependency is a result of the inconsistencies in the signs of the τ/δ and σ/ε relationships which arise due to cyclic loading. By this it is meant that it is possible to incur increases in shear stress associated with a reduction in slip; that is, the negative friction branch F–G in the second quadrant of Figure 5. Moreover, it is possible for reinforcement to develop a compressive stress with an extending strain, for example along path B–C in the second quadrant of Figure 10. To show the influence of the cyclic τ/δ and σ/ε material properties, the mechanics of tension stiffening when unloading is now considered in two distinct phases defined by the reinforcement σ/ε behavior.

### 3.2. Unloading Phase I (Reinforcement in Tension)

**Figure 11:**
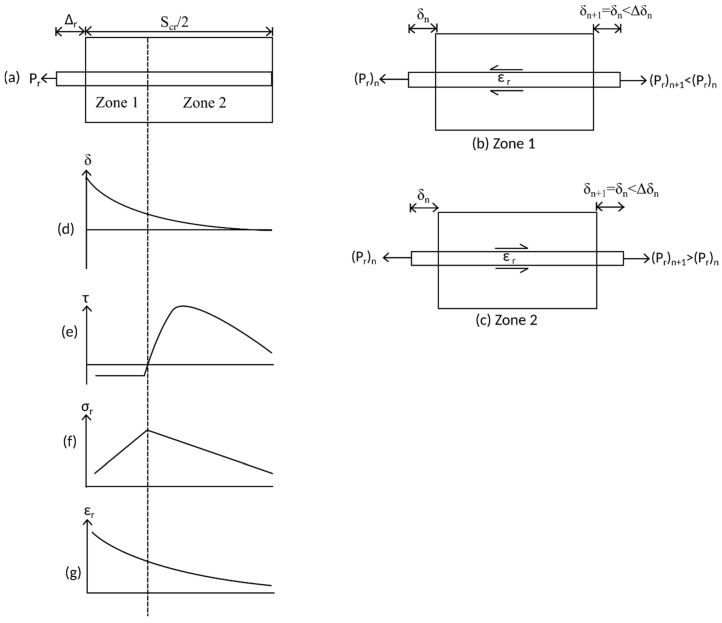
Tension stiffening prism during unloading phase I. (**a**) Beam segment; (**b**) beam element in Zone 1; (**c**) beam element in Zone 2; (**d**) slip distribution; (**e**) bond stress distribution; (**f**) steel stress distribution; and (**g**) steel strain distribution.

The first phase of unloading which takes place along path B–C in Figure 11 is characterized by a reduction in the slip of the bar at the crack face corresponding to a reduction in the applied tensile load. The response of the bar is divided into the two distinct zones shown in Figure 11a.

Consider an element from Zone 1 in Figure 11a shown in Figure 11b. The bar is subjected to a tensile load, but with a force which is reduced from that experienced during initial loading. Assuming the bar had previously yielded so that unloading takes place along branch B–C in Figure 10, a tensile load occurs with an extending strain. The reduction in slip in Zone 1 in Figure 11d is such that the bond stress in Figure 11e is located on the friction branch F–G; that is, in the second quadrant in Figure 5. Thus, from equilibrium across the element in Figure 11b, the bond force acts to increase the load in the bar from the lefthand side to the righthand side and, therefore, the stress in the bar in Figure 11f increases in Zone 1. Although an increase in reinforcement stress occurs, the reinforcement strain as shown in Figure 11g reduces. This behavior occurs as each element of length dx in the PI prism in Figure 11b is assigned a different stress strain relationship which degrades according to the cyclic properties of the reinforcement in Figure 9 which depends on its individual load history. For example, consider an increase in stress between two elements in Zone 1 where the σ/ε relationship of the first element is defined by curve B–C in Figure 10 and, for the second element, by B’–C’. It can be seen in Figure 10 that increasing the stress from *a* on path B–C to *a’* on path B’–C’ results in a reduction in strain. Importantly, as the strain in the reinforcement is an extending strain, the slip strain dδ/dx = ε_r_ − ε_c_ is an extending strain and, therefore, the change in slip across the element δΔ = (dδ/dx)dx results in a reduction in slip.

The behavior characterized in Zone 1 in Figure 11 by an increasing bar stress, but a reducing bar slip, continues until the bond stress is no longer in Quadrant 2 of Figure 5. This may occur either if the change in slip of an element is small enough such that path F’E in Figure 5 is followed, or, if the slip at a given element is greater than that achieved during previous loading, in which case path F’OPQ in Figure 5 is followed. The transition from Zone 1 to Zone 2 which occurs when the bond stress in Figure 11e changes sign can be seen by a reversal of the direction of the bond force B when comparing Figure 11b,c. Elements in Zone 2, such as that shown in Figure 11c are characterized by the bar being pulled with a tensile force and an extending strain and the bond stress resisting the pulling out of the bar such that across each element the bar force reduces. These are identical conditions to that during initial loading as defined by the element in Figure 4c and, hence, convergence on the boundary condition occurs in an identical way. It should also be noted that the unloading phase I behavior when using the new tension stiffening model is almost identical to the behavior obtained from the previous model [32].

### 3.3. Unloading Phase II

**Figure 12:**
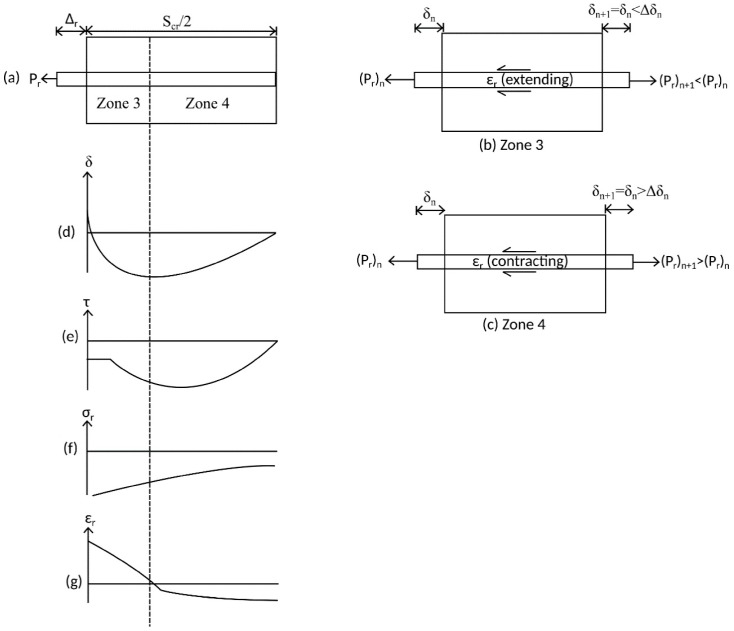
Tension stiffening prism during unloading phase II. (**a**) Beam segment; (**b**) beam element in Zone 3; (**c**) beam element in Zone 4; (**d**) slip distribution; (**e**) bond stress distribution; (**f**) steel stess distribution; and (**g**) steel strain distribution.

The second phase of unloading that is along branch CD in Figure 10 is characterised by the requirement that the bar be pushed in order to further reduce the slip. In this phase, the response of the bar is again divided into two distinct zones as in Figure 12.

An element from Zone 3 in Figure 12a is shown in Figure 12b. In this zone, the bar is subjected to a compressive load and, due to strain hardening, an extending strain; that is, the σ/ε behavior of the element is described by the second quadrant of the reinforcement σ/ε relationship in Figure 12.

Similar to phase I of unloading, in Zone 3 in Figure 12b the reduction in slip due to unloading causes the bond stress to lie in the second or third quadrant of the τ/δ relationship in Figure 5. However in Zone 3 as shown in Figure 12b, equilibrium of the forces over the elements causes a reduction in the bar force and, therefore, stress in Figure 1f. Importantly, as reinforcement has previously strain hardened, in Zone 3 the compressive stress is associated with an extending strain and, thus, the slip reduces along each element as described for Zone 1. The reduction in slip shown in Figure 12d continues until the end of the strain hardening region, that is, at the point where a compressive stress is now associated with a contracting strain (*i.e.*, in the third quadrant of Figure 10).

An element from Zone 4 is shown in Figure 12c. The element is subjected to a compressive stress, a contracting strain, and a bond stress in the second or third quadrant of Figure 5. By equilibrium in Figure 12c, the bond force acts to reduce the bar force and, therefore, stress in the reinforcement towards zero. Moreover, in Zone 4, as the strain in the reinforcement is a contracting strain the slip strain dδ/dx =ε_r_ − ε_c_ has a contracting sense and, therefore, acts over an element length dx to increase the slip Δδ = (dδ/dx)dx towards zero. As shown in Figure 12d, this leads to convergence on the boundary condition that the slip is zero at S_cr_/2.

It should also be noted here that the reversal of slips in Zones 3 and 4 may result in the bar being pushed locally to a slip not previously achieved. That is, slip may result in bond stresses along the negative loading path GHIJK in Figure 5, which implies that damage to the concrete other than due to friction is taking place and this can cause significant reductions in the bond stress transferred for any given slip.

The behavior discussed here is significantly different from the previous work [32]. The new boundary condition used here limits the area of partial interaction, causing the tension stiffening prisms’ behavior to be made of only two distinct zones, whereas in the previous work it features four distinct zones. Additionally, the previous work has two more unloading phases for a total of four unloading phases. This makes the new tension stiffening model significantly less complicated with only two total unloading phases.

### 3.4. Reloading

During reloading along path DE in Figure 9, the mechanics behind each unloading phase already described also applies. A detailed description of the reloading behavior is, therefore, not provided here. However it should be noted that the P_r_/Δ_r_ relationship in Figure 9 can be obtained by seeking the same distributions of slip, bond stress, and reinforcement stress and strain as shown in Figure 11 and Figure 12.

## 4. Cyclic Segmental Analysis between Adjacent Cracks

Having now described the derivation of the reinforcement P_r_/Δ_r_ relationship, these can be directly incorporated into the segmental analysis, described in Figure 3, to simulate the unloading and reloading M/θ relationship between adjacent cracks in Figure 2a. The derivation of the M/θ relationship during initial loading, along path OABC in Figure 2, has been described above with the aid of Figure 3 and, hence, will not be repeated.

### 4.1. Unloading Partial Depth Cracking

Following the commencement of unloading, along path CD in Figure 2, where the bottom reinforcement is unloading but remains in tension, the segmental analysis can be applied in an identical way to that during initial loading. However, when determining the stresses and forces in Figure 3c,d, cyclic constitutive relationships should be used to allow for unloading of both the concrete in compression and the compression reinforcement. The application of the segmental analysis can continue as presented in Figure 3 until the bottom reinforcement develops a compressive stress at point C in Figure 9. At this point, and in order to maintain equilibrium, the uncracked concrete in compression in Figure 3c must move into tension.

### 4.2. Full Depth Cracking

**Figure 13:**
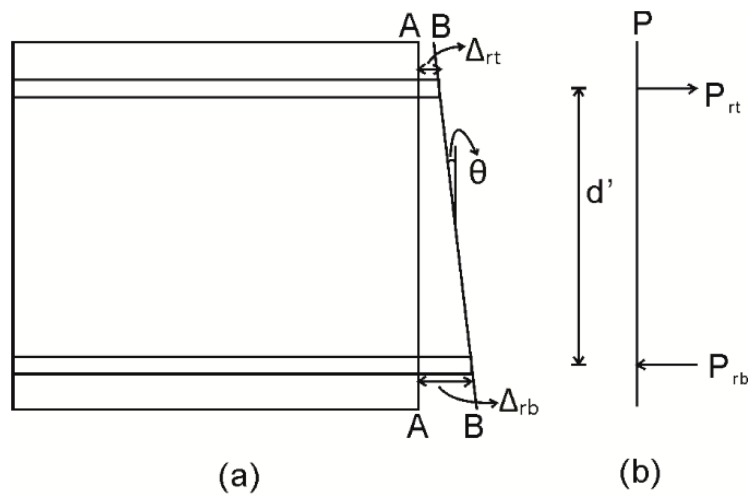
Analysis when segment is cracked full depth. (**a**) Half beam segment; and (**b**) load profile.

The full depth crack results in the mechanism shown in Figure 13 where the segment is cracked to full depth and only the layers of compression and tension reinforcement are interacting. In this case, the P_r_/Δ_r_ relationship developed for the bottom reinforcement still applies and a new P_r_/Δ_r_ relationship is required for the top reinforcement.

When cracked to full depth, the analysis proceeds by imposing a slip Δ_r_ on the layer of reinforcement which is unloading; in the case depicted in Figure 13, this is the bottom layer. The corresponding compression force, P_rb_ in Figure 13, developed in the reinforcement, can then be determined from branch CD in Figure 9. To maintain equilibrium P_rb_ = P_rt_ in Figure 13, and knowing P_rt_, the corresponding slip of the top layer of reinforcement can be determined from path OAB in Figure 9, which is its equivalent for this top reinforcement. Knowing the slip of both the top and bottom layer of the reinforcement, the corresponding rotation from A–A to B–B in Figure 13 is:

(1) $$\theta =\tan^{-1}\left(\frac{\Delta_{rt}-\Delta_{rb}}{d\prime }\right)$$

This analysis can be continued until the slip of the bottom reinforcement reduces to zero. At this stage, the crack is taken to be closed and full interaction is assumed between the reinforcement and the concrete at the closed crack. The analysis can then continue in an identical way to that depicted in Figure 3, but with a residual load in the compression reinforcement which is equal to the load at zero slip, which is point D in Figure 9.

## 5. Cyclic Segmental Analysis in a Hinge

The segmental analysis presented has to this point been concerned with the incorporation of the PI mechanism associated with tension stiffening and crack opening and, hence, considered the behavior between two adjacent cracks. Let us now consider the PI wedge sliding behavior commonly associated with the formation of a plastic hinge [12,14,15,32,33].

**Figure 14:**
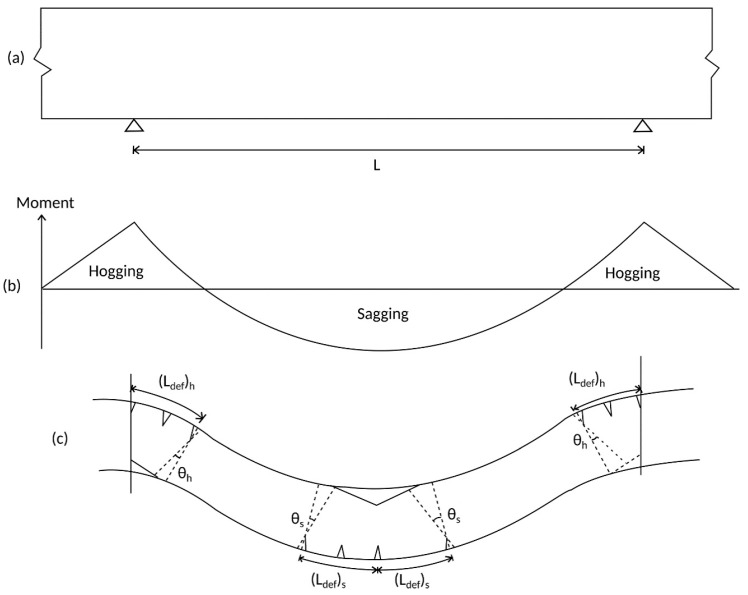
Concentrations of rotation (**a**) RC beam; (**b**) moment distribution; and (**c**) RC beam hinges.

Consider the continuous beam with span length L shown in Figure 14a with the distribution of the applied moment in Figure 14b which causes the deformation shown in Figure 14c. Along the span, concentrations of rotation occur due to concrete cracking and widening, as described by Figure 2, and determined from the segmental approach between adjacent cracks in Figure 3. Additionally, within the span in Figure 14 exists four concentrations of rotation [12] due to concrete cracking and concrete softening; two of these concentrations occur in the hogging region and are of length (L_def_)_h_ and undergo total rotations of θ_h_, and two occur in the sagging region and are of length (L_def_)_s_ and undergo total rotations of θ_s_.

These concentrations of rotation in Figure 14 may be considered as hinges of length L_def_ for the purpose of quantifying rotational behavior following the commencement of concrete softening. It should also be noted that these lengths L_def_ are also the plastic hinge lengths which are commonly used to quantify the ultimate deflection and moment redistribution of RC members and are usually quantified in an empirical manner [23,24,25,26,27,28].

### Multiple Cracking Analysis

**Figure 15:**
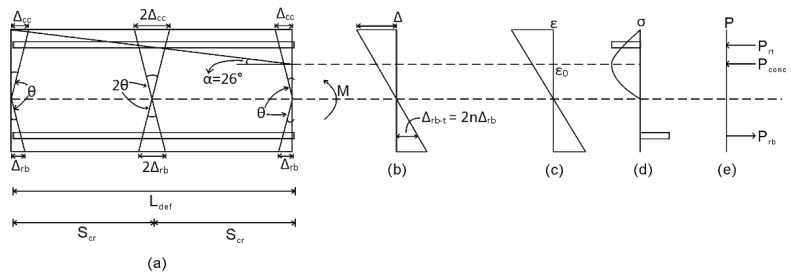
Multiple crack analysis. (**a**) Beam segment; (**b**) displacement profile; (**c**) strain profile; (**d**) stress profile; and (**e**) load profile.

To undertake a segmental analysis where concrete softening is taking place, the half segment length L_def_ in Figure 3 should be of sufficient length such that L_def_ encompasses the total deformation of the PI softening wedge as shown in Figure 14 and enlarged in Figure 15; this requires the consideration of hinges with multiple cracks as in Figure 15. The deformation length L_def_ can be determined from the depth of the concrete softening region and as in Figure 15a is related to depth of the softening region by the angle α of the wedge which can be taken as 26° [33,36]. It should be noted that, for the purpose of this analysis, the softening region is defined by concrete strains exceeding the strain at peak stress ε_0_ in Figure 15c.

For analysis, a total displacement from A–A to B–B in Figure 15b is imposed on the segment end. In the tension region in Figure 15a, slip of the reinforcement Δ_rb_ occurs at each crack face resulting in a total slip of Δ_rb-t_ = 2nΔ_rb_, where n is the number of cracks encompassed by the softening wedge. The load developed in the reinforcement in Figure 15e can then be determined from the P_r_/Δ_r_ relationship in Figure 9 where the load P_rb_ in Figure 15e arises due to the slip from a single crack face Δ_rb_. In the compression region, the analysis is identical to that applied between two cracks. However to allow for the formation and failure of the concrete softening wedge, a size-dependent stress-strain relationship derived from the mechanics of shear friction theory by Chen *et al.* [36] is applied. That is, the stress-strain relationship for the concrete in Figure 15 should be that derived from a material test on a specimen of length 2L_def-analysis_ where Chen *et al.* [36] has proposed the following equation to convert the stress strain behavior extracted empirically from a specimen of length 2L_def-test_ to that of a specimen with a size 2L_def-analysis_.

(2) $$\varepsilon_{Ldef-analysis}=\left(\varepsilon_{Ldef-test}-\frac{\sigma_{a}}{E_{c}}\right)\frac{L_{def-test}}{L_{def-analysis}}+\frac{\sigma_{a}}{E_{c}}$$

where, in Equation (2), ε_Ldef-analysis_ is the strain require for analysis converted from the strain ε_Ldef-test_ extracted from a test on a specimen of total length 2L_def-test_ when a load σ_a_ is applied and E_c_ is the secant modulus of the concrete.

The ability to simulate multiple cracks within one concrete wedge, as presented here, is mainly due to the new tension stiffening analysis used and is considered an improvement over the previous work [32] which could not do so, as it assumes only a single crack for the entire length of the member. The use of size-dependent stress-strain relationship for concrete also presents an improvement to the analysis as the previous work [32] which used a simplified shear friction model and which has limited applicability due to the limited definition of the required material properties.

## 6. Comparison with Test Results

**Figure 16:**
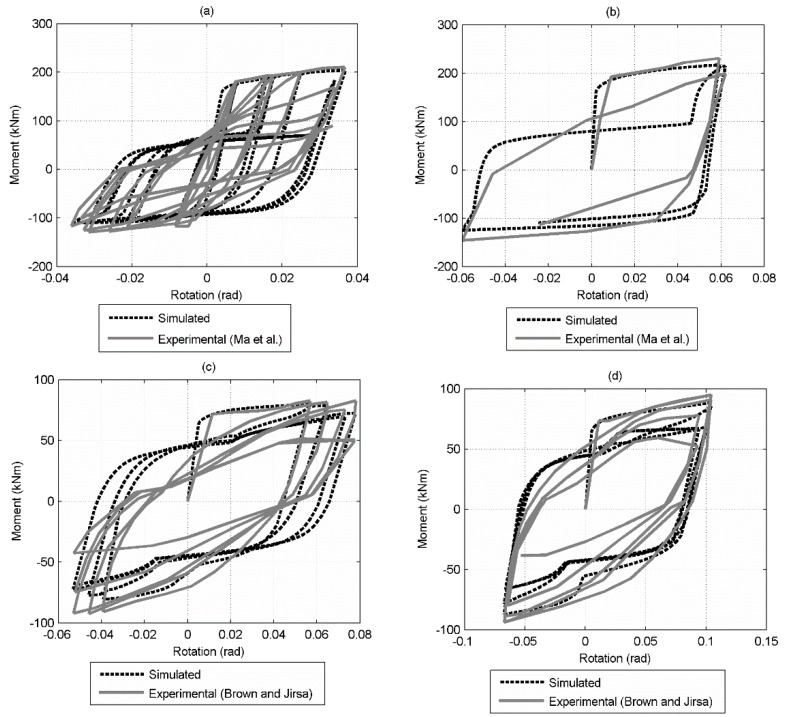
Comparison to experimental results (**a**) Beam R-1; (**b**) Beam R4; (**c**) Beam 88-35-RV5-60; and (**d**) Beam 88-35-RV10-60.

As an example of the application of the segmental M/θ approach, it has been used to predict the moment rotation response of beams tested by Ma *et al.* [37] and Brown and Jirsa [38] in Figure 16. The tests conducted by Ma *et al.* [37] in Figure 16a,b were carried out on cantilever beams of span 1.79 m, effective depth 335 mm and an average concrete compressive strength of 32 MPa. The tests conducted by Brown and Jirsa [38] in Figure 16c,d were carried out on cantilever beams, with spans of 1.52 m and an average concrete compressive strength of 33 MPa. The full details of the beams are given in Table 1 and Table 2.

From Figure 16, it can be seen that the segmental approach generally provides good agreement with the experimental observations. The approach captures the peak of each cycle with good accuracy and reasonably follows the trend of each cycle when the section is fully cracked.

### Material Properties for Analysis

It should be noted that several generic material models were used in the analysis presented in Figure 16. In these models, namely the concrete stress strain relationship proposed by Martínez-Rueda and Elnashai [39], the reinforcement stress strain relationship proposed by Menegotto and Pinto [40] and the bond stress-slip relationship of Eligehausen *et al.* [4] all include factors to degrade the material properties with cyclic loading and, hence, will influence the results presented in Figure 16. It should be noted that these material models are only examples of what could be used in the analysis and, being generic, any material models can be used in the segmental approach.

## 7. Conclusions

The behaviour of reinforced concrete under cyclic loading at all load levels is governed by the local partial interaction mechanisms along reinforcement-to-concrete and concrete-to-concrete sliding planes. These partial interaction behaviors define the formation and widening of cracks in the tension region, as well as tension stiffening in the tension region, and in the compression region, the formation and failure of concrete softening wedges. In this paper, a new segmental moment rotation approach for the cyclic loading of beams has been developed which directly simulates these partial interaction behaviors. For the development of this moment rotation approach, the mechanics of the load slip behavior of reinforcement embedded in concrete under cyclic loading has been derived.

## Figures and Tables

**Table 1:** Summary of beam specimens.

Reference	R-1	R-4	88-35-RV5-60	88-35-RV10-60
Type	Cantilever beam	Cantilever beam	Cantilever beam	Cantilever beam
Beam dimension (mm)	229 × 407	229 × 407	254 × 457	254 × 457
Beam length (mm)	1587.5	1587.5	1520	1520
Amount of tensile bar	4	4	2	2
Amount of compression bar	3	3	2	2
Size of tensile bar (mm)	19.05	19.05	25.4	25.4
Size of compression bar (mm)	15.88	15.88	25.4	25.4

**Table 2:** Material properties.

Beam	R-1	R-4	88-35-RV5-60	88-35-RV10-60
Concrete strength (MPa)	34.96	30.2	33.09	33.09
Concrete elastic modulus (MPa)	28282	26269	27036	27036
Steel reinforcement yield strength (MPa)	451.61	451.61	317.16	317.16

## Notations

The following notations are used in this manuscript:

(L_def_)_h_	Length of deformation in the hogging section of the beam
(L_def_)_s_	Length of deformation in the sagging section of the beam
(P_c_)_n_	Load acting on concrete in element n
(P_r_)_n_	Load acting on reinforcement bar in element n
(P_r_)_n+1_	Load acting on reinforcement bar in element n + 1
∆cc	Deformation due to concrete crushing
∆δ_n_	Change in slip for element n
A_c_	Area of concrete
A_r_	Area of reinforcement bar
B	Bond force
d’	Distance between the top and bottom reinforcement bar
d_NA_	Depth of neutral axis
dδ/dx	Slip-strain
E_c_	Modulus of concrete
E_r_	Modulus of reinforcement bar
L_def_	Length of deformation
L_def-test_	Length of deformation from the experimental compression test of concrete cylinder
L_per_	Perimeter of reinforcement bar
M	moment
P	Load profile
P_c_	Load acting on concrete
P_conc_	Load acting on concrete
P_r_	Load acting on reinforcement bar
P_rb_	Load acting on bottom reinforcement bar
P_rt_	Load acting on top reinforcement bar
S_cr_	Crack spacing
Δ	Deformation profile
δ	Slip of reinforcement bar
δ_n_	Slip of reinforcement bar in element n
δ_n+1_	Slip of reinforcement bar in element n + 1
Δ_r_	Slip of reinforcement bar at the crack face
Δ_rb_	Slip of bottom (tensile) reinforcement bar at the crack face
Δ_rb-t_	Total slip of bottom (tensile) reinforcement bar in multiple crack situation
L_def-analysis_	Length of deformation determined from the tension stiffening analysis
Δ_rt_	Slip of top (compression) reinforcement bar at the crack face
ε	Strain profile
ε_c_	Strain of concrete
ε_ct_	Cracking strain of concrete
ε_Ldef-analysis_	Concrete strain from the experimental compression test of concrete cylinder
ε_r_	Strain of reinforcement bar
θ	Rotation of beam segment
θ_h_	Rotation of beam segment in the hogging section of the beam
θ_s_	Rotation of beam segment in the sagging section of the beam
σ	Stress profile
σ_a_	Stress applied on concrete
σ_r_	Stress applied on reinforcement bar
τ	Bond stress

## References

[1] Bertero V.V., Bresler B. Seismic behavior of reinforced concrete framed structures. Proceedings of the Fourth World Conference on Earthquake Engineering.

[2] Viwathanatepa S., Popov E.P., Bertero V.V. (1979). Effects of Generalized Loadings on Bond of Reinforcing Bars Embedded in Confined Concrete.

[3] Ciampi V., Eligehausen R., Bertero V.V., Popov E.P. (1982). Analytical Model for Concrete Anchorages of Reinforcing Bars under Generalized Excitations.

[4] Eligehausen R., Popov E.P., Bertero V.V. (1982). Local Bond Stress-Slip Relationship of Deformed Bars under Generalized Excitations.

[5] Gupta A.K., Maestrini S.R. (1990). Tension stiffness model for reinforced concrete bars. J. Struct. Eng..

[6] Choi C.K., Cheung S.H. (1996). Tension stiffening model for planar reinforced concrete members. Comput. Struct..

[7] Marti P., Alvarez M., Kaufmann W., Sigrist V. (1998). Tension chord model for structural concrete. Struct. Eng. Int. J. Int. Assoc. Bridg. Struct. Eng..

[8] Yuan H., Teng J.G., Seracino R., Wu Z.S., Yao J. (2004). Full-range behavior of FRP-to-concrete bonded joints. Eng. Struct..

[9] Haskett M., Oehlers D.J., Mohamed Ali M.S. (2008). Local and global bond characteristics of steel reinforcing bars. Eng. Struct..

[10] Yankelevsky D.Z., Jabareen M., Abutbul A.D. (2008). One-dimensional analysis of tension stiffening in reinforced concrete with discrete cracks. Eng. Struct..

[11] Muhamad R., Ali M., Oehlers D.J., Griffith M. (2012). The tension stiffening mechanism in reinforced concrete prisms. Adv. Struct. Eng..

[12] Visintin P., Oehlers D.J., Wu C., Haskett M. (2012). A mechanics solution for hinges in RC beams with multiple cracks. Eng. Struct..

[13] Knight D., Visintin P., Oehlers D.J., Jumaat M.Z. (2013). Incorporating Residual Strains in the Flexural Rigidity of RC members with Varying Degrees of Prestress and Cracking. Adv. Struct. Eng..

[14] Oehlers D.J., Chen J., Ibell T.J. (2014). Simulating reinforced concrete members. Part 1: Partial interaction properties. Proc. Inst. Civ. Eng..

[15] Oehlers D.J. (2014). Simulating reinforced concrete members. Part 2: Displacement-based analyses. Struct. Build..

[16] Zhang T., Visintin P., Oehlers D.J., Griffith M.C. (2014). Presliding Shear Failure in Prestressed RC Beams. II: Behavior. J. Struct. Eng..

[17] Zhang T., Oehlers D.J., Visintin P. (2014). Shear Strength of FRP RC Beams and One-Way Slabs without Stirrups. J. Compos. Constr..

[18] Branson D.E. (1977). Deformation of Concrete Structures.

[19] Gossman J.S. (1980). Simplified Computations for Effective Moment of Inertia Ie and Minimum Thickness to Avoid Deflection Computations. ACI J..

[20] Sakai K., Yakuta Y. (1980). Moment-Curvature Relationships of Reinforced Concrete Members Subjected to Combined Bending and Axial Force. ACI J. Proc..

[21] Al-Zaid R., Al-Shaikh A.H., Abu-Hussein M.M. (1991). Effect of Loading Type on the Effective Moment of Inertia of Reinforced Concrete Beams. ACI Struct. J..

[22] Bischoff P.H. (2005). Reevaluation of Deflection Prediction for Concrete Beams Reinforced with Steel and Fiber Reinforced Polymer Bars. J. Struct. Eng..

[23] Baker A.L.L. (1956). Ultimate Load Theory Applied to the Design of Reinforced and Prestressed Concrete Frame.

[24] Sawyer H.A. Design of Concrete Frames for Two Failure States. Proceedings of the International Symposium on the Flexural Mechanics of Reinforced Concrete.

[25] Corley G.W. (1966). Rotation capacity of reinforced concrete beams. J. Struct. Eng..

[26] Mattock A.H. (1967). Discussion of “rotational capacity of reinforced concrete beams” by WDG Corley. J. Struct. Div..

[27] Priestley M., Park R. (1987). Strength and ductility of concrete bridge columns under seismic loading. ACI Struct. J..

[28] Panagiotakos T.B., Fardis M.N. (2001). Deformations of reinforced concrete members at yielding and ultimate. ACI Struct. J..

[29] Yang Z.J., Chen J. (2005). Finite element modelling of multiple cohesive discrete crack propagation in reinforced concrete beams. Eng. Fract. Mech..

[30] Yang Z.J., Su X.T., Chen J.F., Liu G.H. (2009). Monte Carlo simulation of complex cohesive fracture in random heterogeneous quasi-brittle materials. Int. J. Solids Struct..

[31] Haskett M., Oehlers D.J., Mohamed Ali M.S., Wu C. (2009). Rigid body moment–rotation mechanism for reinforced concrete beam hinges. Eng. Struct..

[32] Visintin P., Oehlers D., Wu C., Griffith M.C. (2012). The reinforcement contribution to the cyclic behaviour of reinforced concrete beam hinges. Earthq. Eng. Struct. Dyn..

[33] Visintin P., Oehlers D. (2013). A Mechanics Based Hinge Analysis for Reinforced Concrete Columns. J. Struct. Eng..

[34] Tastani S.P., Pantazopoulou J. (2015). Implications of yield penetration on confinement requirements of r.c. wall elements. Earthq. Struct..

[35] Tastani S.P., Pantazopoulou J. (2013). Yield penetration in seismically loaded anchorages: Effects on member deformation capacity. Earthq. Struct..

[36] Chen Y., Visintin P., Oehlers D.J., Alengaram U.J. (2014). Size-Dependent Stress-Strain Model for Unconfined Concrete. J. Struct. Eng..

[37] Ma S.M., Bertero V.V., Popov E.P. (1976). Experimental and Analytical Studies on the Hysteretic Behavior of Reinforced Concrete Rectangular and T-Beams.

[38] Brown R., Jirsa J. (1971). Reinforced concrete beams under load reversals. ACI J. Proc..

[39] Martínez-Rueda J., Elnashai A. (1997). Confined concrete model under cyclic load. Mater. Struct..

[40] Menegotto M., Pinto P.E. Method of analysis for cyclically loaded reinforced concrete plane frames including changes in geometry and non-elastic behavior of elements under combined normal force and bending. Resistance and Ultimate Deformability of Structures Acted on by Well-Defined Repeated Loads, Proceedings of the IABSE Symposium.

